# Biological Age Is Associated with the Active Use of Nutrition Data

**DOI:** 10.3390/ijerph15112431

**Published:** 2018-11-01

**Authors:** Kyu-Tae Han, Dong Wook Kim, Seung Ju Kim, Sun Jung Kim

**Affiliations:** 1 Research and Analysis Team, National Health Insurance Service Ilsan Hospital, Goyang 10444, Korea; kthan.phd@gmail.com (K.-T.H.); kimdw2269@gmail.com (D.W.K.); 2 Institute of Health Services Research, Yonsei University College of Medicine, Seoul 03722, Korea; 3 Department of Nursing, College of Nursing, Eulji University, Seongnam 13135, Korea; seungju.phd@gmail.com; 4 Department of Health Administration and Management, Soonchunhyang University, Asan 31538, Korea

**Keywords:** biological age, nutrition facts, health information, patient awareness

## Abstract

*Purpose* Biological age (BA) has recently emerged as a substitute for chronological age (CA), and many subjects seek to optimally control their BA. However, in South Korea, no study has adequately explored factors that affect BA, although individual health management is essential to preventing chronic diseases. In the present study, we focus on the use of health information, in particular nutrition facts, to control BA. *Methods* We used data from the Korea National Health and Nutrition Examination Surveys (2010–2015; 26,914 eligible participants) using BA and age differences as outcome variables. We used multiple linear regression to explore the relationship between the use of nutrition data and differences in BA after adjusting for covariates. In addition, we used multiple linear regression to examine subgroup differences in such relationships. *Results* 12.8% of males and 27.5% of females used nutrition facts when deciding which foods to purchase. The more attention paid to such facts, the lower the BA and BA differences in both males and females (males: β = −2.646, females: β = −2.787, *p* < 0.05, for BA; males: β = −1.181, females, β = −2.161, *p* < 0.05, for BA differences). However, BA differences were more significant in subjects with chronic disease, obesity, and/or a family history of chronic disease. *Conclusion* High-level awareness of and active use of nutrition facts permitted effective self-management in preventing chronic disease and improving BA, particularly in subjects at higher risk for chronic disease. Thus, considering nutrition facts when deciding what to purchase is important.

## 1. Introduction

Aging is characterized by a gradual decline in physiological function and by morphological changes and is usually assessed in terms of chronological age (CA) [1]. However, by the development of socioeconomic and public health aspects, disease patterns have changed over time. The recent increase in lifestyle-related diseases such as obesity, hypertension, and diabetes mellitus has rendered lifestyle modifications essential to preventing and managing chronic diseases [2,3]. Many people began to self-manage their health to prevent chronic diseases including cancer at aging society [4,5]. CA can no more sufficiently reflect physiological function, general health, or overall decline [6]. Thus, an index evaluating the difference between expected health status based on CA and actual health status is needed.

Biological age (BA) is the age indicator which estimated by measuring health status biomarkers, has been used to estimate physiological function, overall health status, and aging [7,8]. BA is a useful index that enables subjects to understand their health status and emphasizes the importance of a healthy lifestyle [9,10,11,12]. The BA is also considered in the predicting of mortality among specific population [13]. BA has attracted increasing attention since 2000, and many of the factors that affect BA are now known. Health behavior such as physical activity and diet affect BA [14,15]. No South Korean study has adequately evaluated BA or the factors that affect BA, although it is important to manage individual health to prevent chronic disease and remain healthy life. In this study, we explored whether attention paid to nutrition facts affects BA.

Beginning in 1995, South Korean law mandated that all processed foodstuffs list nutrition information to inform the making of healthy choices [16]. This prevents many health problems and has been applauded by nutrition and public health professionals. Such labeling protects against worsening health and plays an important role in the self-management of chronic diseases [17,18]. However, better health outcomes are not ensured if nutrition data are not actively used [19]. Thus, we hypothesized that the active use of nutrition information would be associated with healthy life, and explored the association between the use of nutrition information and BA in this study.

## 2. Methods

### 2.1. Study Population

We used data from two Korea National Health and Nutrition Examination Surveys (KNHANES V and VI; 2010–2015). The KNHANES studies are cross-sectional in nature and have been conducted annually since 1998 by the Korea Centers for Disease Control (KCDC) using a stratified, multistage, cluster sampling design. The surveys include three questionnaires: Health Interview Survey, Health Examination, and Nutrition Survey. All participants were interviewed by trained personnel. The overall response rates were 80.8% for KNHANES V and 78.3% for KNHANES VI, resulting in 60,917 respondents in total. Respondents who did not provide data that would have enabled us to calculate BA and those <20 years of age were excluded, as were subjects who did not report their awareness of nutrition facts. We ultimately included 26,914 eligible participants.

### 2.2. Variables

To explore whether BA improves upon active pursuit of nutrition data, we calculated BA by referring to metabolic syndrome status and calculated the difference between BA and CA (outcome variables) [20,21] as follows:

BA in males = −76.0965 + 0.541 × (waist circumstance) + 0.271 × (mean blood pressure) + 0.213 × (fasting blood glucose level) + 0.059 × (triglyceride level) − 0.312 × (high-density lipoprotein cholesterol level) + 0.850 × (age)

BA in females = −66.530 + 0.484 × (waist circumstance) + 0.328 × (mean blood pressure) + 0.303 × (fasting blood glucose level) + 0.080 × (triglyceride level) − 0.282 × (high-density lipoproteins cholesterol level) + 0.601 × (age)

The primary variable of interest was the use of nutrition information. It was defined based on response for three phases question in KNHANES. If respondents answered as “Yes” for first question of “Do you know the nutrition labeling?”, they answered for the next following question by stage: “Do you check the nutrition labeling when you purchase food?” and “Nutrition labeling affect to your decision on purchasing food?” Based on these responses, the use of nutrition information was defined as follows: (1) nonuse (respondent is unaware of the availability of nutrition data), (2) use (respondent is aware of the availability of data but does not check data), or (3) active use (respondent checks nutrition facts and makes informed purchase decisions). If respondents answered “Yes” three times in a low, they was defined “active use”. Other independent variables were age, education level, economic status, household income, body mass index (BMI), any chronic disease, aerobic exercise habits, smoking status, alcohol intake, any family history of chronic disease, survey year, stress level, subjective health status, and average daily energy intake. Subjects were grouped by age as follows: <30 years, 30–39 years, 40–49 years, 50–59 years, and ≥60 years. Subjects were grouped by BMI as follows: BMI < 23 kg/m^2^, underweight or normal; BMI = 23–25 kg/m^2^, overweight; and BMI > 25 kg/m^2^, obese. A personal or family history of chronic disease was defined as at least one of diabetes mellitus, dyslipidemia, or hypertension. The cutoff for weekly aerobic exercise time was 150 min. High-risk drinking was defined as the consumption of more than seven (males) or five (females) drinks on a single occasion at least twice a week. The stress level was defined based on answer for “How often do you feel stressed in your daily life?”. If respondents answered as “Often” or “Very often”, they was defined as “High”. The average daily energy intake was based on that on the day before the survey, using a 24 h recall method; the investigators calculated energy intake using these data.

### 2.3. Statistical Analysis

Descriptive statistics are reported as frequencies with percentages (categorical variables) or as means with standard deviations (continuous variables). We used *t* tests or analysis of variance (ANOVA) to identify relationships between independent variables and BA and BA difference (BA–CA). Finally, we performed multiple linear regression to explore the relationship between the use of nutrition data and BA and BA difference after adjusting for covariates. In addition, we performed subgroup analyses by age, education level, BMI, diagnosis of a chronic disease, and family history of a chronic disease. We evaluated males and females separately. We applied a sampling weight to each participant to be able to generalize the data. SAS version 9.4 (SAS Institute, Cary, NC, USA) was used for all analyses.

## 3. Results

We included 26,914 respondents in this study (males: 11,009, females: 15,905). Table 1 shows the general characteristics of the study population. More than 70% of respondents knew that nutrition facts were available, and 12.8% of males and 27.5% of females actively used these facts when deciding what food to purchase.

Table 2 shows the results of ANOVAs of BA and BA–CA data by independent variable, including the use of nutrition data. The overall average BA and BA–CA were 54.66 and 3.35 in males and 54.03 and 4.02 in females, respectively. Those exhibiting higher nutrition fact use exhibited lower BA and BA–CA (both males and females); the comparisons were statistically significant (all *p* < 0.001). Age exhibited a positive linear association with both BA and BA–CA. Higher socioeconomic status (SES) was inversely associated with BA, but a sex-specific difference was apparent. In addition, those who were obese or overweight exhibited higher BA and BA–CA than others (all *p* < 0.0001). Smoking and frequent drinking of alcohol also exhibited positive linear associations with both BA and BA–CA. In addition, those diagnosed with chronic diseases exhibited significantly higher BA and BA–CA (See Table S1 in Supplementary Materials).

To explore the association between the use of nutrition facts (a measure of health information perception) and BA and BA–CA, we performed linear regression analyses after adjusting for other independent variables. Greater use of nutrition facts was inversely associated with reductions in BA and BA–CA for both males and females (active use, males: β = –2.646, females: β = –2.787, *p* < 0.05; use, males: β = −1.181, females: β = −2.161, *p* < 0.05). However, BA–CA varied significantly by active use status (males: β = −1.695, females: β = −0.817, *p* < 0.05; use, males: β = −0.360, females: β = −0.201, *p* > 0.05). In terms of other covariates, respondents with higher SES had lower BA, and those with high BMI or with chronic diseases had higher BA and BA–CA. Exercise habits, alcohol intake, and smoking status significantly influenced both BA and BA–CA, as did family history of chronic disease and subjective health status (Table 3 and Table S2).

**Figure 1:**
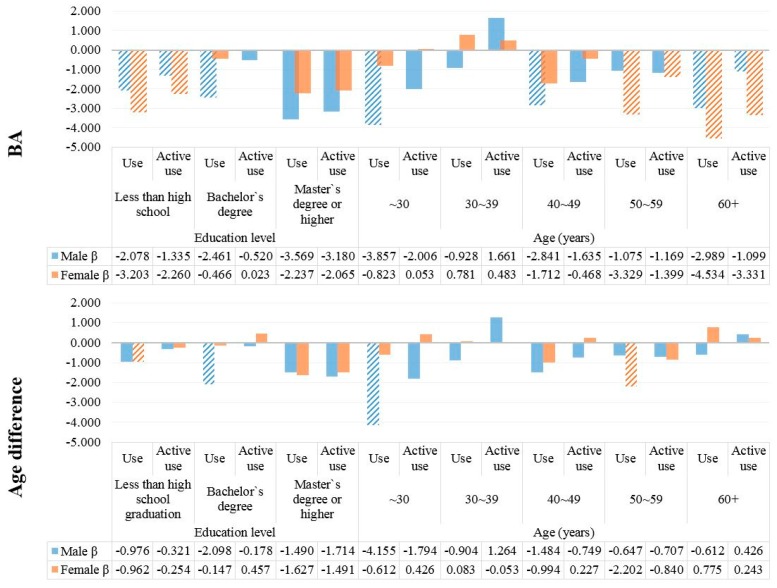
Subgroup linear regression analyses exploring associations between the use of nutrition facts and BA and BA–CA by age and education level. Marked plot mean statistically significant (*p* < 0.05).

**Figure 2:**
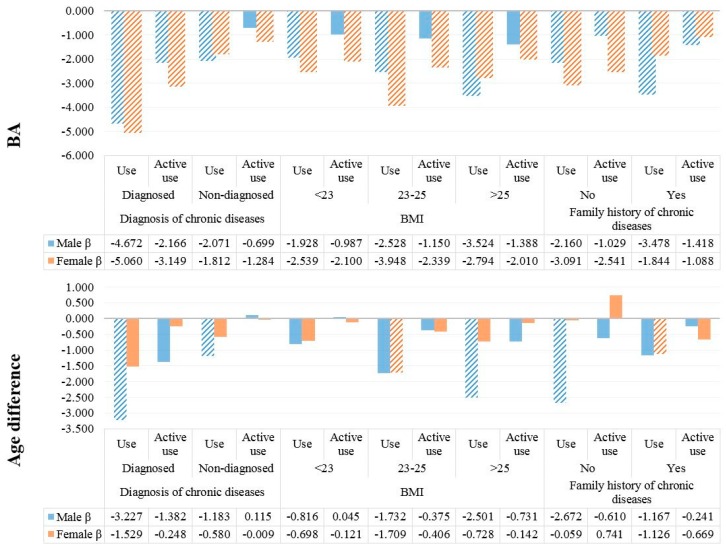
Subgroup linear regression analyses exploring associations between the use of nutrition data and BA and BA–CA by clinical status. Marked plot mean statistically significant (*p* < 0.05).

We also performed subgroup analyses to explore differences in the use of nutrition data and BA or BA–CA by age, education level, chronic disease status, BMI, and family history of chronic disease. In terms of age and education level, the reductions in BA evident upon high-level use of nutrition facts were greater (and similar) in those who were poorly educated and >60 years of age. In terms of clinical status, the reductions in BA evident upon high-level use of nutrition facts were greater in those with higher BMI, with chronic (Figure 1 and Figure 2).

## 4. Discussion

Public health professionals seek primarily to improve overall health worldwide. However, if subjects do not internalize public health advice, nothing will be achieved. This is also true when it comes to nutrition data. Greater use of such data affords better health outcomes, but many may not engage in active use of these data [18]. Thus, we focused on the use of nutrition data that became available (by law) in Korea in 1995 [16]. One of our outcome variables was BA, which is used to evaluate individual health and aging. Differences in lifestyle and physiological status cause BA to vary among subjects with the same CA [6].

Our findings suggest that greater use of nutrition facts improves individual health outcomes, in particular BA and BA–CA, in line with previous findings on the relationship between nutrition data availability and health outcomes in those with obesity or chronic disease [22,23]. Active use of health information improves health outcomes; self-management using nutrition data improves BA, a new indicator of health status. To date, physical activity and diet have primarily been considered to affect BA; the use of nutrition data has not been adequately addressed. Thus, given the emerging concept of BA, the importance of health information, including nutrition facts, cannot be overemphasized.

In terms of the other covariates, most of our findings are in agreement with data from earlier studies. Poorer health behavior, a high BMI, and chronic disease increased BA and BA–CA. Obesity, low-level physical activity, chronic disease, smoking, and alcohol consumption increase BA [24]. In addition, our subgroup analyses showed that the relationships between BA and use of nutrition facts differed by the subgroup variable studied. The improvements in BA and BA–CA evident upon active use of nutrition data were greater in those at a higher risk for increasing BA. Its associations were more significant in those with chronic disease, those who were obese, those with a family history of chronic disease and/or a low education level, and the elderly [25]. Thus, active use of nutrition information would be more helpful in health self-management among relatively vulnerable subjects than the general population. Health care professionals and policymakers must seek to improve public health awareness, particularly in vulnerable populations, emphasizing the positive impact of nutrition awareness on self-management in preventing the development of chronic disease. Our findings will aid in the establishment of health care programs and policies; public awareness is essential. Experts should modify existing programs rather than establish new initiatives.

Our study has several strengths over earlier works. First, the data were representative of the national population, surveyed via health interview, health examination, and a nutrition test. Thus, our results reflect the overall situation in South Korea and may have external validity. Second, we used BA and BA–CA as outcome variables. As society ages, BA may be a more effective indicator of health than CA, because individuals vary in terms of lifestyle and clinical features. Thus, health outcome assessment using nutrition data may become increasingly relevant. However, few studies have treated BA as an outcome variable, and none have done so in the nutrition or public health sphere in South Korea. Thus, we suggest the use of BA as a novel outcome variable. In addition, although nutrition facts became widely available about 10 years ago in South Korea, few studies (none prior to 2010) have explored customer awareness or use of these data. Thus, our data may be useful for designing nutrition programs.

However, our data were derived from cross-sectional (not longitudinal) studies; we thus have no follow-up information on respondents. We cannot infer cause-and-effect relationships. In addition, the use of nutrition data was self-reported; recall bias may have been a factor. In addition, BA and BA–CA used as outcome variables were originally developed to evaluate health and aging in patients with chronic diseases. Therefore, the positive association we describe between reductions in BA and active use of nutrition data may not be generalizable to all types of BA (such as bone or blood vessel age). Moreover, BA was calculated using only data obtained on the survey day, which could have introduced measurement errors. Finally, optimal evaluation of any positive impact of nutrition data availability should include information on how such use actually influences food consumption. We lacked such data.

Despite these limitations, we found that active use of nutrition data improved health outcomes, in particular BA and BA–CA. The improvement in BA was more significant in those with chronic disease, those who were obese, those who were older, and those with a low education level. Health care professionals and policymakers should consider the importance of nutrition data when establishing new programs and should adequately inform the public.

## 5. Conclusions

Our findings suggested that high awareness or active use of nutrition data facilitated self-management in preventing chronic disease and improving BA. In particular, this was more significant in those with chronic disease, those who were obese, those with a family history of chronic disease and/or a low education level, and the elderly. Thus, the importance of nutrition data for making food purchase decisions must be emphasized. Decision makers involved in nutrition policy should consider the activation of nutritional information, not just creating a few scope policies. By doing this, it will be expected that BA will be well managed, and diseases will be prevented.

## Figures and Tables

**Table 1:** General characteristics of the study population by sex.

Variable	Males		Females	
	Frequency	%	Frequency	%
**Use of nutrition data**				
Active use	1127	12.8	4010	27.5
Use	5844	58.8	7298	49.3
Nonuse	4038	28.5	4597	23.2
**Age (years)**				
<30	1274	20.0	1818	17.2
30–39	1777	20.1	2947	20.1
40–49	1911	21.2	2957	21.8
50–59	2117	19.3	3262	19.4
≥60	3930	19.4	4921	21.4
**Education level**				
Less than high school	6187	48.7	10,408	60.6
Bachelor’s degree	4159	45.3	5043	36.4
Master’s degree or higher	663	6.0	454	3.1
**Economic status**				
Unemployed	2922	23.1	8118	48.2
Employed	8087	76.9	7787	51.8
**Household income**				
Low	1939	13.2	3077	16.4
Medium to low	2813	25.6	4089	26.6
Medium to high	3113	31.0	4327	28.7
High	3144	30.2	4412	28.3
**BMI (kg/m^2^)**				
<23	4172	37.5	7802	51.7
23–25	2852	25.4	3465	20.7
>25	3985	37.1	4638	27.6
**Chronic disease**				
Diagnosed	3482	23.3	4694	23.8
Not diagnosed	7527	76.7	11,211	76.2
**Aerobic exercise status**				
Yes	3501	35.1	4070	27.7
No	7508	64.9	11,835	72.3
**Smoking status**				
Smoker	4114	41.4	754	5.7
Ex-smoker	4638	35.8	822	6.0
Nonsmoker	2257	22.8	14,329	88.3
**Alcohol intake**				
Less than twice a week	9023	79.7	15,305	95.4
More than twice a week	1986	20.3	600	4.6
**Family history of chronic disease**				
No	7373	65.1	10,042	62.0
Yes	3636	34.9	5863	38.0
**Survey year**				
2010	2085	16.8	2951	16.8
2011	2031	17.2	2999	17.8
2012	1827	16.7	2781	16.9
2013	1738	16.6	2474	16.2
2014	1594	15.9	2322	15.6
2015	1734	16.8	2378	16.6
**Stress level**				
Low	8633	76.3	11,597	71.4
High	2376	23.7	4308	28.6
**Subjective health status**				
Good	3988	38.0	4629	30.0
Normal	5312	48.1	7882	50.2
Bad	1709	13.8	3394	19.8
**Average daily energy intake ^†^**	2472	13.7	1740	7.7
**Total**	11,009	100.0	15,905	100.0

^†^ The mean and standard deviation of continuous variable. Note: Percentages may not add up to exactly 100%, owing to the rounding off.

**Table 2:** Average BA and BA–CA by independent variable.

Variable	Males						Females					
	BA			Difference (BA–CA)			BA			Difference (BA–CA)		
	Mean	SD	*p*-Value	Mean	SD	*p*-Value	Mean	SD	*p*-Value	Mean	SD	*p*-Value
**Use of Nutrition Data**												
Active use	44.34	20.81	<0.0001	3.15	13.82	0.0128	43.31	18.25	<0.0001	2.48	13.19	0.0371
Use	49.74	21.17		3.55	14.50		49.80	20.59		3.71	13.63	
Nonuse	64.66	18.26		3.12	14.87		70.11	18.52		5.86	14.74	

Adjusted age, education level, economic status, household income, BMI, any chronic disease, aerobic exercise habits, smoking status, alcohol intake, any family history of chronic disease, survey year, stress level, subjective health status, and average daily energy intake.

**Table 3:** Results of linear regression analyses of the association between the use of nutrition data and BA or BA–CA.

Variable	Males						Females					
	BA			Difference (BA–CA)			BA			Difference (BA–CA)		
	β	SE	*p*-Value	β	SE	*p*-Value	β	SE	*p*-Value	β	SE	*p*-Value
**Use of Nutrition Data**												
Active use	−2.646	0.573	<0.0001	−1.695	0.559	0.0025	−2.787	0.374	<0.0001	−0.817	0.365	0.0256
Use	−1.181	0.397	0.003	−0.360	0.386	0.3519	−2.161	0.338	<0.0001	−0.201	0.326	0.5385
Nonuse	Ref	-	-				Ref	-	-			

Adjusted age, education level, economic status, household income, BMI, any chronic disease, aerobic exercise habits, smoking status, alcohol intake, any family history of chronic disease, survey year, stress level, subjective health status, and average daily energy intake.

## Supplementary Materials

The following are available online at http://www.mdpi.com/1660-4601/15/11/2431/s1, Table S1. Average BA and BA–CA by independent variable. Table S2. Results of linear regression analyses of the association between the use of nutrition data and BA or BA–CA.
