# Supplementary material for: Biological Age Is Associated with the Active Use of Nutrition Data
Source: Int J Environ Res Public Health. 2018 Nov 1;15(11):2431. doi: 10.3390/ijerph15112431 (PMC6266208; doi:10.3390/ijerph15112431)
Supplement: Supplementary file 1 [file ijerph-15-02431-s001.pdf]

Table S1. Average BA and BA–CA by independent variable.

Variable	Males						Females					
	BA			Difference (BA–CA)			BA			Difference (BA–CA)		
	Mean	SD	P-value	Mean	SD	P-value	Mean	SD	P-value	Mean	SD	P-value
**Use of nutrition data**												
Active use	44.34	20.81	<0.0001	3.15	13.82	0.0128	43.31	18.25	<0.0001	2.48	13.19	0.0371
Use	49.74	21.17		3.55	14.50		49.80	20.59		3.71	13.63	
Nonuse	64.66	18.26		3.12	14.87		70.11	18.52		5.86	14.74	
**Age (years)**												
<30	22.82	14.03	<0.0001	-1.33	13.07	<0.0001	25.90	11.21	<0.0001	1.62	10.61	<0.0001
30–39	39.57	14.83		4.60	14.56		36.87	12.86		1.96	12.49	
40–49	50.64	15.78		6.35	15.42		47.98	15.05		3.61	14.66	
50–59	61.12	15.64		6.50	15.55		60.15	15.00		5.83	14.63	
≥60	70.28	13.44		1.16	13.25		74.28	15.03		5.19	14.54	
**Education level**												
Less than high school	61.87	18.95	0.2873	3.53	15.00	0.5499	62.58	19.88	<0.0001	5.66	14.75	<0.0001
Bachelor's degree	44.26	21.34		2.95	14.04		37.69	16.39		1.00	11.58	
Master's degree or higher	52.60	18.61		4.28	13.73		39.62	15.87		0.11	11.19	
**Economic status**												
Unemployed	59.72	24.30	<0.0001	1.64	13.78	0.1040	57.09	22.81	<0.0001	4.38	13.90	0.0833
Employed	52.83	20.18		3.97	14.80		50.85	20.92		3.65	13.91	
**Household income**												
Low	65.35	20.05	<0.0001	1.78	14.63	0.9760	69.12	19.73	<0.0001	5.64	14.72	<0.0001
Medium to low	55.29	21.90		3.07	14.71		55.18	22.66		5.33	15.17	
Medium to high	50.94	20.82		3.76	14.31		49.11	20.46		3.74	12.97	
High	51.19	20.63		4.18	14.59		47.28	19.43		1.95	12.66	
**BMI (kg/m^2)**												
<23	46.75	21.72	<0.0001	-5.20	11.72	<0.0001	43.34	19.25	<0.0001	-2.19	10.36	<0.0001
23–25	55.96	20.16		3.61	12.10		58.68	19.14		5.03	12.32	
>25	62.01	19.47		12.12	13.58		68.54	19.05		13.72	14.49	
**Chronic disease**												
Diagnosed	69.70	16.31	<0.0001	7.58	15.57	<0.0001	73.24	17.02	<0.0001	9.40	15.88	<0.0001
Not diagnosed	47.70	20.10		1.40	13.65		45.99	18.82		1.77	12.32	
**Aerobic exercise status**												
Yes	51.66	22.11	<0.0001	2.55	14.26	<0.0001	51.05	20.96	<0.0001	3.21	13.54	<0.0001
No	56.06	21.16		3.73	14.70		55.06	22.42		4.30	14.02	
**Smoking status**												
Smoker	52.09	21.31	<0.0001	4.75	15.60	<0.0001	50.19	23.01	<0.0001	6.48	16.02	<0.0001
Ex-smoker	60.37	19.53		3.16	14.02		47.82	22.73		4.18	13.69	
Nonsmoker	47.59	22.97		1.20	13.42		54.59	21.97		3.88	13.79	
**Alcohol intake**												
Less than twice a week	54.57	21.36	<0.0001	2.22	13.69	<0.0001	54.38	22.02	0.0288	3.95	13.74	0.0025
More than twice a week	55.07	22.46		8.49	17.14		45.18	22.90		5.93	17.72	
**Family history of chronic disease**												
No	54.77	21.82	0.1641	2.31	14.16	0.0014	54.27	22.68	0.0144	3.65	13.91	0.0002
Yes	54.43	21.04		5.46	15.15		53.63	21.12		4.66	13.89	
**Survey year**												
2010	55.09	21.94	<0.0001	4.29	14.91	<0.0001	53.33	21.68	0.0241	4.60	12.86	0.0204
2011	54.63	21.45		2.76	14.91		54.30	22.86		3.84	14.43	
2012	54.67	20.72		2.22	13.24		55.20	22.50		4.14	14.59	
2013	52.61	21.57		3.05	15.03		52.85	21.72		3.95	13.71	
2014	54.50	21.03		3.16	14.11		53.08	21.43		3.12	13.74	
2015	56.36	22.43		4.60	14.91		55.35	22.23		4.36	14.02	

**Stress level**												
Low	55.66	21.61	0.5851	2.95	14.32	0.5982	54.67	21.75	0.2352	3.94	13.57	0.9373
High	51.03	21.01		4.81	15.38		52.33	23.02		4.24	14.79	
**Subjective health status**												
Good	50.36	21.95	<0.0001	1.53	13.09	<0.0001	48.63	21.10	<0.0001	2.46	12.61	0.0018
Normal	55.37	20.80		4.17	14.81		53.18	21.49		3.97	13.85	
Bad	62.49	20.50		5.07	16.52		63.40	22.04		6.28	15.37	
**Total**	**54.66**	**21.56**		**3.35**	**14.57**		**54.03**	**22.12**		**4.02**	**13.91**	

Table S2. Results of linear regression analyses of the association between the use of nutrition data and BA or BA–CA.

Variable	Males						Females					
	BA			Difference (BA–CA)			BA			Difference (BA–CA)		
	β	SE	P-value	β	SE	P-value	β	SE	P-value	β	SE	P-value
**Use of nutrition data**												
Active use	-2.646	0.573	<0.0001	-1.695	0.559	0.0025	-2.787	0.374	<0.0001	-0.817	0.365	0.0256
Use	-1.181	0.397	0.003	-0.360	0.386	0.3519	-2.161	0.338	<0.0001	-0.201	0.326	0.5385
Nonuse	Ref	-	-				Ref	-	-			
**Age (years)**												
<30	Ref	-	-				Ref	-	-			
30–39	13.160	0.522	<0.0001	2.548	0.489	<0.0001	9.348	0.362	<0.0001	-1.043	0.336	0.002
40–49	24.095	0.536	<0.0001	3.835	0.508	<0.0001	17.950	0.439	<0.0001	-1.801	0.418	<0.0001
50–59	32.679	0.541	<0.0001	3.171	0.521	<0.0001	25.165	0.463	<0.0001	-3.441	0.446	<0.0001
≥60	40.918	0.563	<0.0001	-1.670	0.551	0.0025	34.221	0.533	<0.0001	-7.582	0.511	<0.0001
**Education level**												
Less than high school	-0.205	0.650	0.7526	-0.029	0.616	0.9627	2.602	0.589	<0.0001	2.320	0.563	<0.0001
Bachelor's degree	-0.799	0.610	0.1906	-0.336	0.575	0.5594	0.264	0.557	0.6353	0.772	0.522	0.1395
Master's degree or higher	Ref	-	-				Ref	-	-			
**Economic status**												
Unemployed	Ref	-	-				Ref	-	-			
Employed	-1.335	0.386	0.0006	-0.970	0.360	0.0072	-0.843	0.231	0.0003	-0.483	0.229	0.0352
**Household income**												
Low	1.156	0.511	0.0239	-0.426	0.485	0.3800	1.565	0.426	0.0002	0.663	0.412	0.1080
Medium to low	-0.022	0.417	0.9581	-0.059	0.406	0.8844	0.728	0.350	0.0374	1.130	0.338	0.0008
Medium to high	-0.012	0.384	0.9756	-0.002	0.369	0.9961	0.275	0.277	0.3215	0.502	0.267	0.0606
High	Ref	-	-				Ref	-	-			
**BMI (kg/m^2)**												
<23	Ref	-	-				Ref	-	-			
23–25	7.924	0.330	<0.0001	8.100	0.317	<0.0001	7.135	0.288	<0.0001	7.085	0.275	<0.0001
>25	16.225	0.350	<0.0001	16.593	0.334	<0.0001	15.777	0.357	<0.0001	15.971	0.349	<0.0001
**Chronic disease**												
Diagnosed	6.303	0.437	<0.0001	5.802	0.420	<0.0001	7.856	0.400	<0.0001	6.594	0.396	<0.0001
Not diagnosed	Ref	-	-				Ref	-	-			
**Aerobic exercise status**												
Yes	-1.577	0.324	<0.0001	-1.365	0.307	<0.0001	-1.430	0.258	<0.0001	-1.046	0.253	<0.0001
No	Ref	-	-				Ref	-	-			
**Smoking status**												
Smoker	Ref	-	-				Ref	-	-			
Ex-smoker	-1.207	0.353	0.0007	-1.770	0.343	<0.0001	-1.836	0.769	0.0171	-2.103	0.717	0.0034
Nonsmoker	-1.863	0.371	<0.0001	-1.742	0.356	<0.0001	-1.817	0.670	0.0068	-1.832	0.629	0.0037
**Alcohol intake**												
Less than twice a week	Ref	-	-				Ref	-	-			
More than twice a week	2.968	0.430	<0.0001	3.205	0.420	<0.0001	2.087	1.237	0.0918	2.509	1.216	0.0392
**Family history of chronic disease**												
No	Ref	-	-				Ref	-	-			
Yes	0.702	0.380	0.0647	0.979	0.361	0.0068	0.757	0.274	0.0058	0.881	0.271	0.0012
**Survey year**												
2010	-1.097	0.554	0.0481	-0.733	0.524	0.1619	0.361	0.391	0.3564	0.901	0.372	0.0157
2011	-1.398	0.579	0.0159	-1.124	0.558	0.044	-0.443	0.436	0.3101	-0.004	0.427	0.9934
2012	-2.256	0.550	<0.0001	-1.912	0.537	0.0004	0.726	0.537	0.1771	1.197	0.526	0.023
2013	-2.135	0.527	<0.0001	-1.840	0.505	0.0003	-0.805	0.401	0.0449	-0.250	0.398	0.5306
2014	-0.962	0.516	0.0624	-0.879	0.500	0.0794	-0.325	0.399	0.4152	-0.113	0.391	0.7721
2015	Ref	-	-				Ref	-	-			

**Stress level**												
Low	Ref	-	-				Ref	-	-			
High	0.107	0.375	0.7755	0.466	0.357	0.1926	-0.078	0.312	0.8039	0.103	0.306	0.7357
**Subjective health status**												
Good	-2.977	0.508	<0.0001	-2.733	0.500	<0.0001	-1.444	0.354	<0.0001	-1.136	0.339	0.0008
Normal	-1.263	0.490	0.0101	-1.050	0.480	0.0288	-0.838	0.329	0.011	-0.506	0.317	0.1103
Bad	Ref	-	-				Ref	-	-			
**Average daily energy intake**	-0.011	0.014	0.4596	0.003	0.014	0.8378	-0.039	0.020	0.0490	-0.006	0.019	0.7480
